# Differential effects of a post-anthesis heat stress on wheat (*Triticum aestivum* L.) grain proteome determined by iTRAQ

**DOI:** 10.1038/s41598-017-03860-0

**Published:** 2017-06-14

**Authors:** Yufeng Zhang, Jiajia Pan, Xiuwen Huang, Dandan Guo, Hongyao Lou, Zhenghong Hou, Meng Su, Rongqi Liang, Chaojie Xie, Mingshan You, Baoyun Li

**Affiliations:** Beijing Key Laboratory of Crop Genetic Improvement/Key Laboratory of Crop Heterosis & Utilization, Ministry of Education, College of Agronomy, China Agricultural University, Beijing, 100193 China

## Abstract

Heat stress, a major abiotic stressor of wheat (*Triticum aestivum* L.), often results in reduced yield and decreased quality. In this study, a proteomic method, Tags for Relative and Absolute Quantitation Isobaric (iTRAQ), was adopted to analyze the protein expression profile changes among wheat cultivar Jing411 under heat stress. Results indicated that there were 256 different proteins expressed in Jing411 under heat stress. According to the result of gene annotation and functional classification, 239 proteins were annotated by 856 GO function entries, including growth and metabolism proteins, energy metabolism proteins, processing and storage proteins, defense-related proteins, signal transduction, unknown function proteins and hypothetical proteins. GO enrichment analysis suggested that the differentially expressed proteins in Jing411 under heat stress were mainly involved in stimulus response (67), abiotic stress response (26) and stress response (58), kinase activity (12), and transferase activity (12). Among the differentially expressed proteins in Jing411, 115 were attributed to 119 KEGG signaling/metabolic pathways. KEGG pathway enrichment analysis in Jing411 showed that heat stress mainly affected the starch and sucrose metabolism as well as protein synthesis pathway in the endoplasmic reticulum. The protein interaction network indicated that there were 8 differentially expressed proteins that could form an interaction network in Jing411.

## Introduction

Global temperature has been increasing since the beginning of the century, and this trend is predicted to continue into the future^1^. Wheat is one of the most highly cultivated cereals in the world, and like other cultivated crops, wheat production is significantly affected by abiotic stress especially at high temperature during the grain filling stage. Therefore, study of the genetic and molecular mechanisms underlying heat tolerance in wheat is of great importance for heat-resistant molecular breeding.

Recently, proteomics approaches, such as two-dimensional gel electrophoresis/mass spectrometry (2-DE/MS), have emerged as powerful methods for identifying and quantifying the large number of proteins in biological samples^2–4^. The influence of heat stress on wheat grain was revealed by studies of wheat proteome by 2-DE/MS^3–5^, and many proteins that belonged to a variety of protein families and were involved in various biological processes were differentially expressed in response to heat stress. Skylas *et al*.^6^ found many heat shock proteins that ranged in molecular mass from 15 to 30 kDa, which increased immediately following a 3-day heat treatment from 15 to 17 days post-anthesis (DPA), and these proteins were differentially expressed in the heat-susceptible cultivar Wyuna and the heat-tolerant cultivar Fang. Yang *et al*.^7^ reported 11 spots changed in a gliadin protein fraction when high temperatures were applied from anthesis to 10 DPA. Seven were identified as α-gliadins, one as a γ-gliadin and three as low-molecular-weight glutenin subunits (LMW-GSs). Laino *et al*.^8^ studied changes in the metabolic protein profiles of durum wheat under heat stress, revealing differentially expressed proteins mainly involved in glycolysis, carbohydrate metabolism, and the stress response. Dupont *et al*.^9, 10^ studied the response of a sampling of proteins in each gluten protein class during endosperm development and reported increases in high-molecular-weight glutenin subunits (HMW-GSs) and α-gliadins, and a decrease in a major LMW-GS in response to temperature. Majoul *et al*.^11^ identified 42 proteins that were enzymes involved in different metabolic pathways of plants, such as granule-bound starch synthase and glucose-1-phosphate adenyl transferase, which are involved in the starch synthesis pathway, and β-amylase, which is involved in carbohydrate metabolism. Hurkman *et al*.^12^ revealed that high temperature accelerated the development of wheat grain and high temperature reduced the accumulation of grain metabolism-related proteins but induced accumulation of proteins related to storage and defense, perhaps as a self-protective mechanism to cope with heat stress. Through analysis of early developmental changes in the metabolic protein profile of wheat grain, Nadaud *et al*.^13^ found that heat shock proteins (HSPs) were expressed throughout the early grain development stages. Majoul *et al*.^11^ reported that levels of HSPs as well as proteins that defend against reactive oxygen species (ROS) and desiccation increased in flour when developing grain was exposed to an extended high temperature from anthesis to maturity. Yang *et al*.^7^ found peroxiredoxins, late embryogenesis abundant proteins, α-amylase inhibitor, and serine proteinase inhibitor abundance changes in wheat grain subjected to drought or heat stress, but the abundance of HSPs and 14-3-3 proteins changed only under heat stress. Moreover, some proteins are difficult to identify by MS because they are of very low abundance or other reasons.

Heat stress affects the synthesis of grain proteins, cellular metabolism, carbohydrate metabolism, and the activities of critical enzymes involved in transcription and translation, thus disturbing the normal development of grain. 2-DE and MS are commonly used to analyze changes in the proteome. To date, isobaric tags for relative and absolute quantitation (iTRAQ) has not been applied to study changes in protein expression in wheat grain at heat stress. iTRAQ is a new, powerful and simultaneous, iTRAQ technology developed by Applied Biosystems Incorporation (ABI) in 2004^14^. The method of absolute and relative quantitative studies of four samples is based on the iTRAQ reagent. The iTRAQ reagent is an amine-labeled isobaric that is linked to the amino acid N-terminal and lysine side chains. In the mass spectrum, the same protein in the different samples labeled with any iTRAQ reagent showed the same mass-to-charge ratio. In the tandem mass spectrometry, the signal ions show peaks with different mass-to-charge ratios (114 to 117). Therefore, according to the height and area of the peaks, the quantitative information of the protein can be obtained^15^.

Compared with two-dimensional electrophoresis, iTRAQ has the following advantages: (1) strong separating power and large analysis scope; (2) reliable qualitative analysis, which can determine the molecular weight and structural information of each component simultaneously; (3) highly sensitive mass spectrometry; (4) rapid analysis and effective separation; (5) a highly automatic process; and (6) effective detection of cytoplasmic, membrane-bound, nuclear, and extracellular proteins. The iTRAQ technique can be used to detect proteins with low abundance and strongly alkaline proteins, as well as proteins smaller than 10 kDa or larger than 200 kDa. iTRAQ has been extensively applied in disease marker detection, cell difference analysis, drug development, and cancer research. In the present study, iTRAQ was used to study the influence of heat stress on protein expression in wheat grain, with the goal of identifying genes closely related to heat stress and providing a theoretical basis for research on wheat heat tolerance.

## Results

### Changes in grain traits at high temperature

In addition to genetic factors, the quality traits of wheat grain are influenced by environmental conditions such as heat stress, which reduces wheat yield and lowers quality. In the present work, the thousand kernel weight, grain length and width of wheat cultivar Jing411 were determined after heat stress. Wheat grain subjected to heat stress showed significant narrowing, while their length was unchanged (Fig. S1a,c,d), compared with wheat grain from wheat subjected to normal growth conditions. These results suggest that heat stress may affect grain filling by reducing grain size, ultimately reducing a thousand grain weight of wheat. As seen in Fig. S1b, high temperature reduced the SDS-sedimentation value of wheat grain, indicating that heat stress led to deterioration of wheat quality.

### Grain protein identification by iTRAQ and functional annotation

iTRAQ-based differential protein identification was performed using grain from wheat cultivar Jing411 after high temperature treat (T) and normal (CK) treatment, revealing 2,493 proteins that had quantitative information in the tags of each channel (Table S1, Sheet 1). The sequences of the quantified proteins were extracted in batches from the UniProt Knowledgebase (UniProtKB). The correlation coefficients (r) of the protein expression levels in the three replicates from the high temperature treatment and control treatment groups were greater than 0.7, confirming the reliability of the results (Fig. S2).

Proteins were screened for differential expression based on standard criteria: expression ratio >1.2 and P-value < 0.05. After high temperature treatment, 256 proteins were differentially expressed in wheat cultivar Jing411, including 126 up-regulated proteins and 130 down-regulated proteins (Table S1, Sheet 2).

### Sequence alignment and functional annotation of differentially expressed proteins

The similarity between the protein sequences of wheat cultivar Jing411 and those in the NCBI nr database ranged from 40–100%, with most target proteins having a sequence similarity of more than 94% (Fig. S3). The mapping function in Blast2GO (Version 2.8.0) was used to extract Gene Ontology (GO) terms associated with the hits obtained after a BLAST search, retrieving 1,801 GO terms associated with the sequences of 237 differentially expressed proteins (91.51%). Appropriate GO terms were assigned to target proteins in Blast2GO based on comprehensive consideration of the similarity between the target sequences and aligned sequences, GO term reliability, and GO structure (cyclic or not). The annotation analysis assigned 414 GO terms to 149 proteins with an average GO level of 5.169 (Fig. S4).

After the first round of annotation, annotation restrictions were relaxed to obtain more functional annotation information for target proteins that were not annotated with hits in BLAST. Through the supplementary annotation, 239 differentially expressed proteins were annotated by 856 GO terms (Table S2, protein2GO).

### GO annotation of differentially expressed proteins

**Figure 1:**
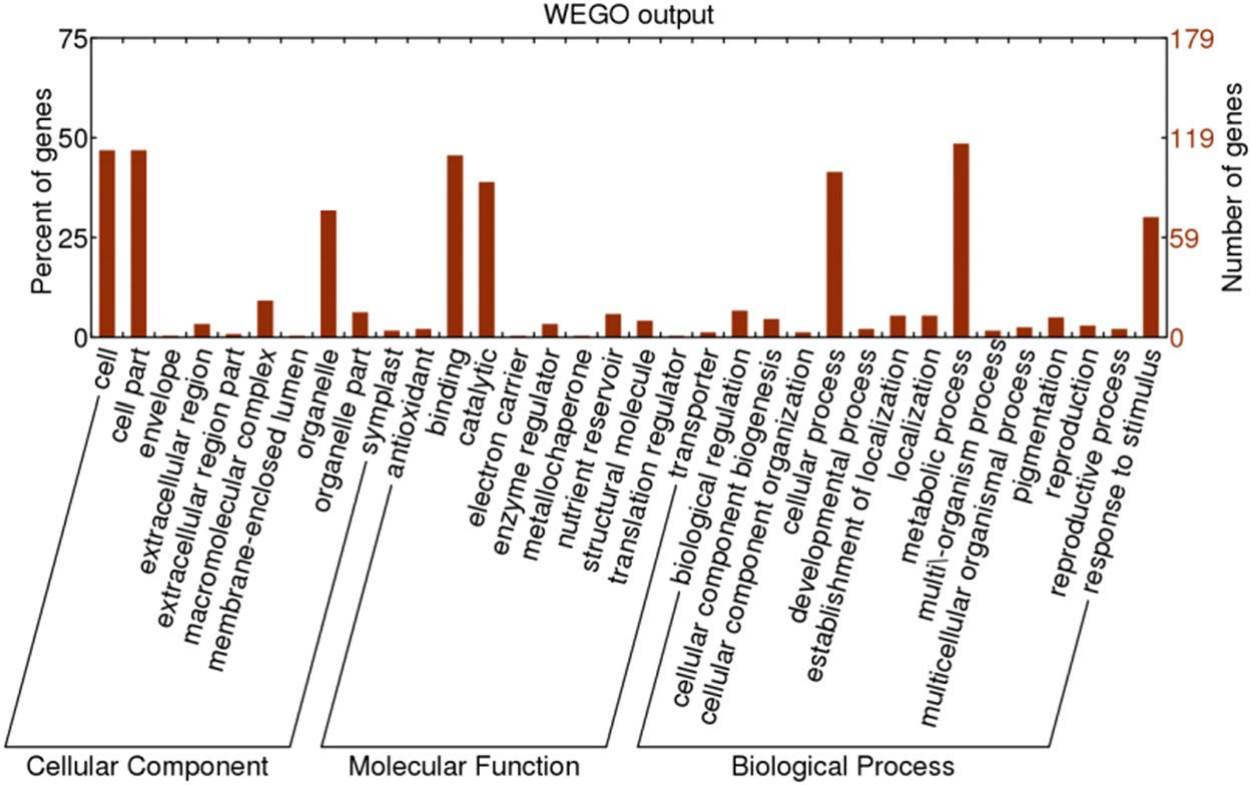
GO annotation terms of differentially expressed proteins at GO level 2 in wheat cultivar Jing411.

In terms of cellular components, 181 proteins were annotated, mainly including membrane proteins and organelle-forming proteins (Table S2, CC); in terms of molecular function, 275 proteins were annotated, mainly including ATP-binging, DNA-binding and ribosome-binding proteins, as well as proteins involved in kinase activity (Table S2, MF); and 400 proteins were annotated in terms of biological process, indicating that they were mainly involved in starch metabolism, sucrose metabolism, stress response, transcription, and redox (Table S2, BP). Differentially expressed proteins in the grain of wheat cultivar Jing411 were classified at GO level 2 by various functions (Fig. 1), as follows: (1) biological process: response to stimuli (67 proteins), cellular processes (94), and metabolic processes (122); (2) molecular function: catalyzing activities (93), binding function (109), regulating enzyme activity (9), and structure molecular activity (10); (3) cellular component: cell (108), membrane (25), and organelle (52) components; (Table S2, Level2_BP, Level2_CC Level2_MF). Some of the differentially expressed proteins possessed multiple functions.

### GO enrichment analysis for differentially expressed proteins

**Figure 2:**
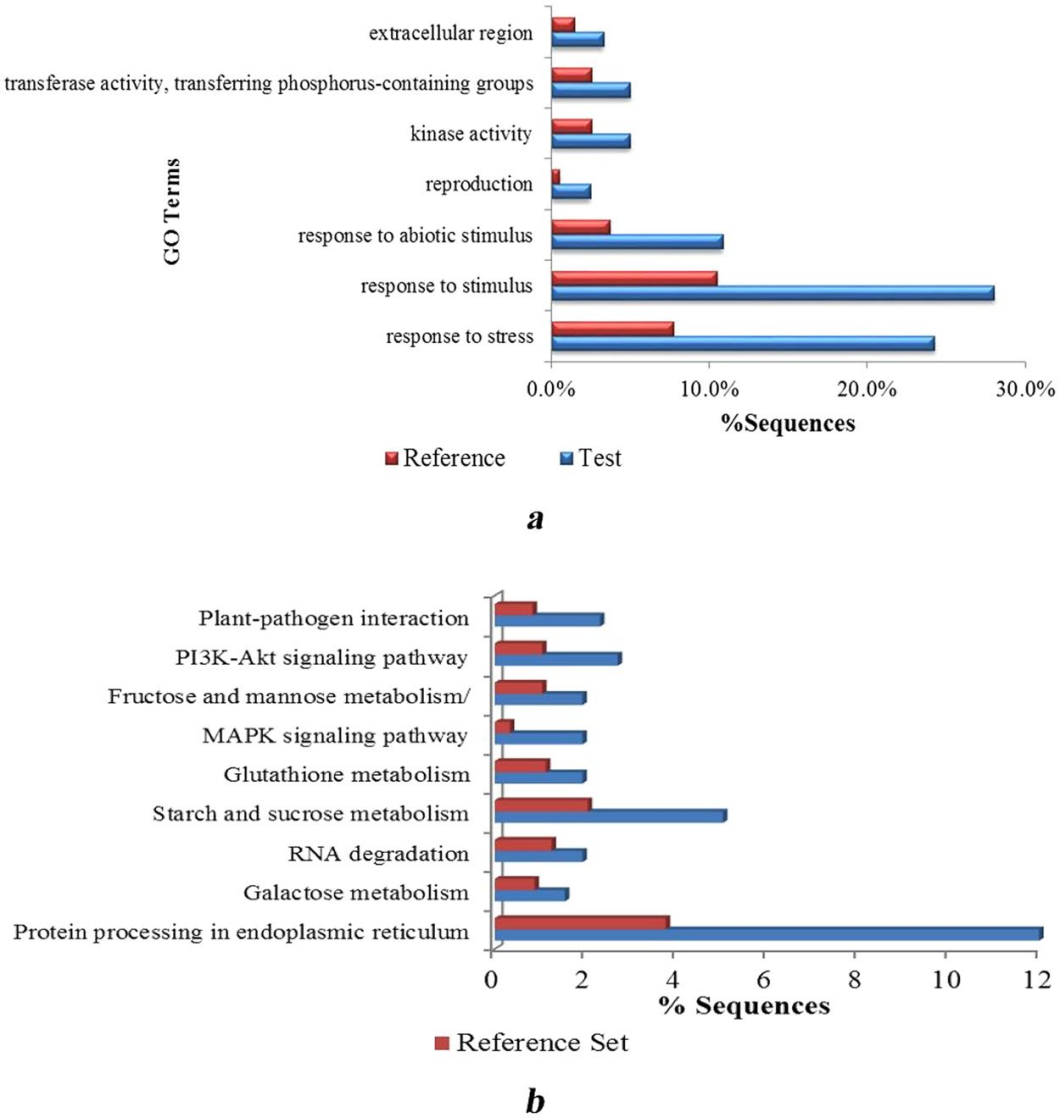
GO enrichment analysis and KEGG pathway enrichment analysis of differentially expressed proteins. (**a**) Significantly enriched GO terms in the differentially expressed proteins of wheat cultivar Jing411. (**b**) KEGG pathway enrichment analysis for differentially expressed proteins in wheat cultivar Jing411.

The significantly enriched GO terms were analyzed under the background of all the identified proteins using Fisher’s exact test to identify significantly enriched GO terms: response to abiotic stimuli (26 proteins), response to stimuli (67), stress response (58), kinase activity (12), and transferase activity (12) (Fig. 2a; Table S2, GO_enrich). GO enrichment analysis showed that the differentially expressed proteins mainly participated in responses to stimuli and stresses.

### KEGG pathway annotation and KEGG pathway enrichment analysis of differentially expressed proteins

KEGG pathway enrichment analysis, with the identified proteins as the background set, determined significantly enriched pathways in the set of differentially expressed proteins by Fisher’s exact test, thus determining the main relevant metabolic and signal transduction pathways. Analysis of the set of 115 differentially expressed proteins extracted 119 associated KEGG signaling/metabolic pathways (Table S3, TopMapStat), among which 85 significantly enriched pathways were obtained by KEGG pathway enrichment analysis (Fig. 2b; Table S3, Enrichment).

### Analysis of protein-protein interaction networks

**Figure 3:**
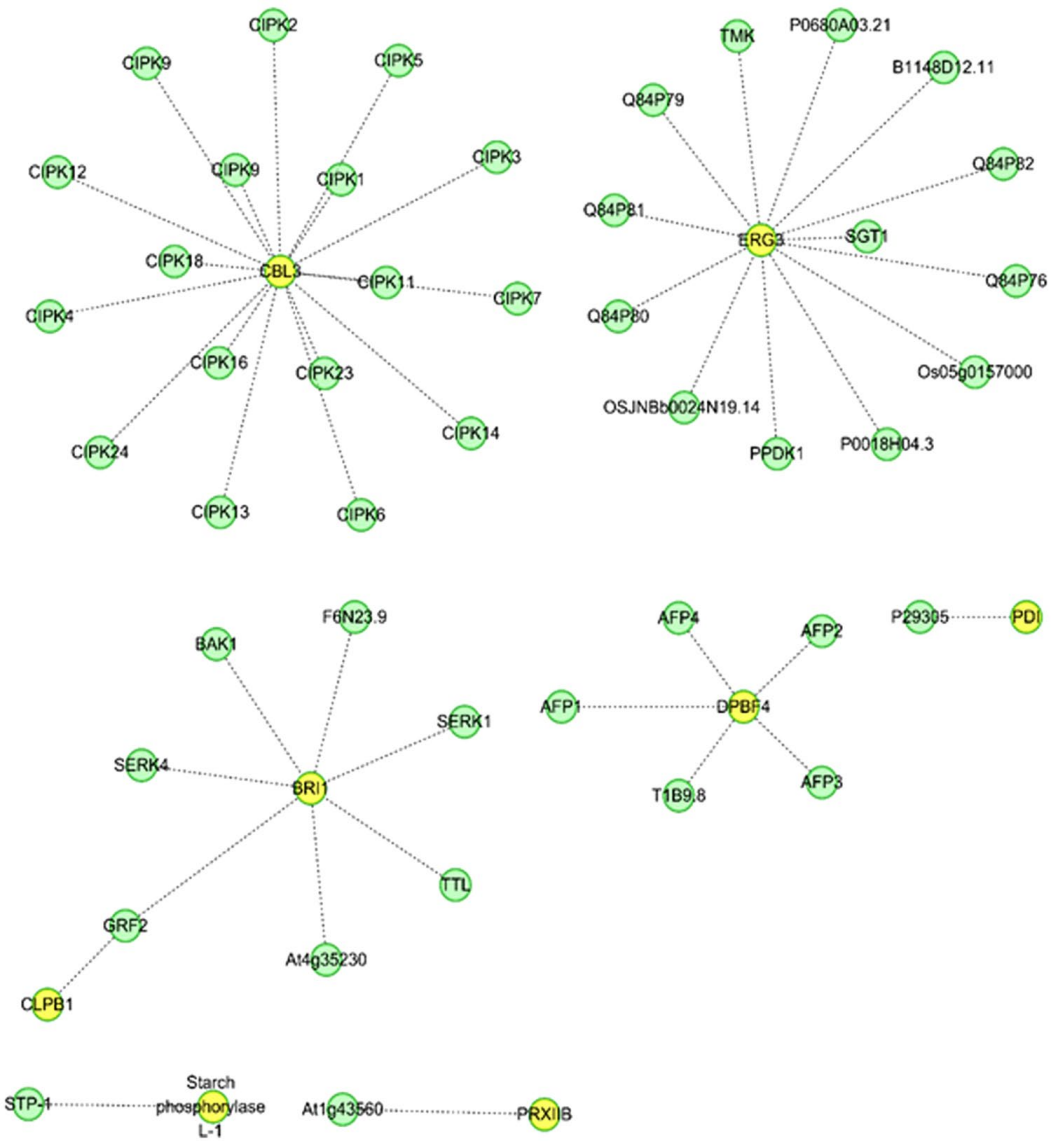
Interaction networks of the differentially expressed proteins in wheat cultivar Jing411. Yellow nodes denote differentially expressed proteins, whereas green nodes denote proteins interacting directly with the differentially expressed proteins. Calcineurin B-like 3 (CBL3), C-5 sterol desaturase (ERG3), Brassinosteroid receptor 1 (BRI1), DPBF4, Protein disulfide isomerase (PDI), Chaperone protein (CLPB1), starch phosphorylase L-1, and Peroxiredoxin B (PRXIIB).

Interactions between proteins and the resulting interaction networks reveal protein functions. The interactions between target proteins and other proteins were determined by searching for the GeneSymbols of the identified differentially expressed proteins in the IntAct database, after which interaction networks were generated using Cytoscape software. Eight differentially expressed proteins, including Calcineurin B-like 3 (CBL3), C-5 sterol desaturase (ERG3), brassinosteroid receptor 1 (BRI1), DPBF4, protein disulfide isomerase (PDI), Chaperone protein (CLPB1), starch phosphorylase L-1, and Peroxiredoxin B (PRXIIB) in the grain of wheat cultivar Jing411, were involved in known interaction networks and might perform critical regulatory functions under heat stress (Fig. 3).

## Discussion

### Analysis of differentially expressed proteins

In this study, proteins related to energy metabolism, growth and development, and stresses were shown to be involved in the response to heat stress during the development of wheat grains, offering a resource for future studies. Using iTRAQ, we identified 256 differentially expressed proteins from wheat cultivar Jing411 cultivated at normal and high temperature. We found that *HSP90* significantly increased and *LMW*-*GS*, adenosine diphosphoglucose pyrophosphorylase (*ADPG*-*PPase*) and starch branching enzyme IIb (*SBEIIb*) significantly decreased according to real-time quantitative methods at 15 and 20 DPA under heat stress (Fig. S5). These results are consistent with the differential expression of proteins identified by iTRAQ. After the high temperature treatment, protein disulfide isomerase (PDI), Myb-related proteins, 40S ribosomal protein, 60S ribosomal protein, and DnaJ protein were up-regulated in wheat cultivar Jing411, whereas ADPG-PPase, pyrophosphate-fructose-6-phosphate 1-phosphotransferase (PFP), and sucrose synthase were down-regulated.

Disulfide bonds, formed by the covalent cross-linking of sulfhydryl groups at the cysteine side chain, play a critical role in stabilizing the three-dimensional structure of proteins, retarding the folding processes of many proteins^15^. The lumen of the endoplasmic reticulum (ER) contains a series of oxidoreductases that catalyze the formation of disulfide bonds, of which PDI is the predominant type in wheat^16, 17^. PDI catalyzes the formation of disulfide bonds between proteins and repairs incorrectly formed disulfide bonds^18^. Heat stress may affect the synthesis and assembly of proteins. In wheat, PDI might be up-regulated during heat stress to correct mistakes in protein folding. Oxidative stress following high temperature may adversely influence membrane systems. Abnormal endoplasmic reticulum function affects protein synthesis and disturbs cellular metabolism. PDI plays a regulatory role in the domain located in the endoplasmic reticulum, and this regulation can link the redox potential in the endoplasmic reticulum to the redox pathway in the cytoplasm, which may play an important role in heat stress.

Myb transcription factor, a member of the largest plant transcription factor family, is involved in cell division, cell cycle regulation, responses to hormones and environmental factors. In addition, it also has an important regulatory effect on plant secondary metabolism and leaf morphology^19^. Hurkman *et al*.^12^ identified changes in 40S and 60S ribosomal proteins as a response to high temperature treatment at 10–20 DPA. Ribosomal proteins influence the transcription efficiency and stability of ribosomes and participate in DNA repair, apoptosis, and gene expression regulation. The results of this study suggest that PFP, Myb-related proteins, 40S and 60S ribosomal proteins, and PDI are up-regulated in wheat under heat stress to maintain the normal functions of cells and enhance heat tolerance.

DnaJ proteins can activate and control the ATP enzyme activity of HSP70 to influence several processes in cells, including folding of newly formed proteins, endocytosis, transport of polypeptides through the plasma membranes of organelles, regulation of various stress responses, and targeting of pre-degraded proteins^20–22^. The number of DnaJ proteins varies with species: yeast have 22^21^, humans have 41^23^, and *Arabidopsis* have 89^24^. Several DnaJ genes have been identified and functionally annotated in rice^25, 26^, but few reports on wheat DnaJ proteins exist.

ADPG-PPase participates in the first step of starch synthesis using sucrose as a substrate. ADPG-PPase is a crucial enzyme for starch synthesis, and its gene expression and activity have a direct influence on the starch content in endosperm. Hence, variation in ADPG-PPase expression influences starch accumulation in wheat grain, impacting wheat yield^27^. Weigelt *et al*.^28^ showed that reducing starch content enhanced the metabolic activity of mitochondria in peas and led to excessive production of reactive oxygen species, thus disturbing normal metabolic activity.

PFP is a cytosolic enzyme that is widely expressed in plant tissues and catalyzes the transformation between fructose-6-phosphate and fructose-1,6-diphosphate by phosphorylation and dephosphorylation. PFP actively participates in producing necessary intermediate products, providing energy for respiration, inducing CO_2_ accumulation and synthesis of exportable carbohydrates in crassulacean acid metabolism (CAM) plants^29^. In addition, PFP acts in the gluconeogenic direction and supplies the ppi needed for sucrose degradation, which is favorable for the synthesis and storage of sucrose and starch in some plant tissues (e.g., carrot and tomato fruits). Laino *et al*.^8^ also found PFP down-regulation under heat stress using two-dimensional electrophoresis. Sucrose synthase plays a critical role in plant growth and development. Leaf photosynthates products are largely transported to “library” organs in the form of sucrose, and sucrose synthase is one of the key enzymes necessary for sucrose to enter a variety of metabolic pathways^30^. Down-regulation of sucrose synthase indirectly leads to carbohydrate production, eventually reducing wheat yield amd quality.

HSPs play an important role in the response to heat stress and participate in protein folding and assembly. HSPs are produced as a response to heat stress. In the present study, HSP90, HSP101, HSP26, HSP40, heat shock cognate 70 kDa protein, mitochondrial HSP70, and heat shock 70 kDa protein were up-regulated in wheat cultivar Jing411; some of these proteins were identified by Laoino *et al*.^8^ using two-dimensional electrophoresis and showed the same trends in expression in previous studies when under heat stress. There is a wide variety of heat shock proteins, and each protein specific function requires further study.

### GO enrichment analysis

**Figure 4:**
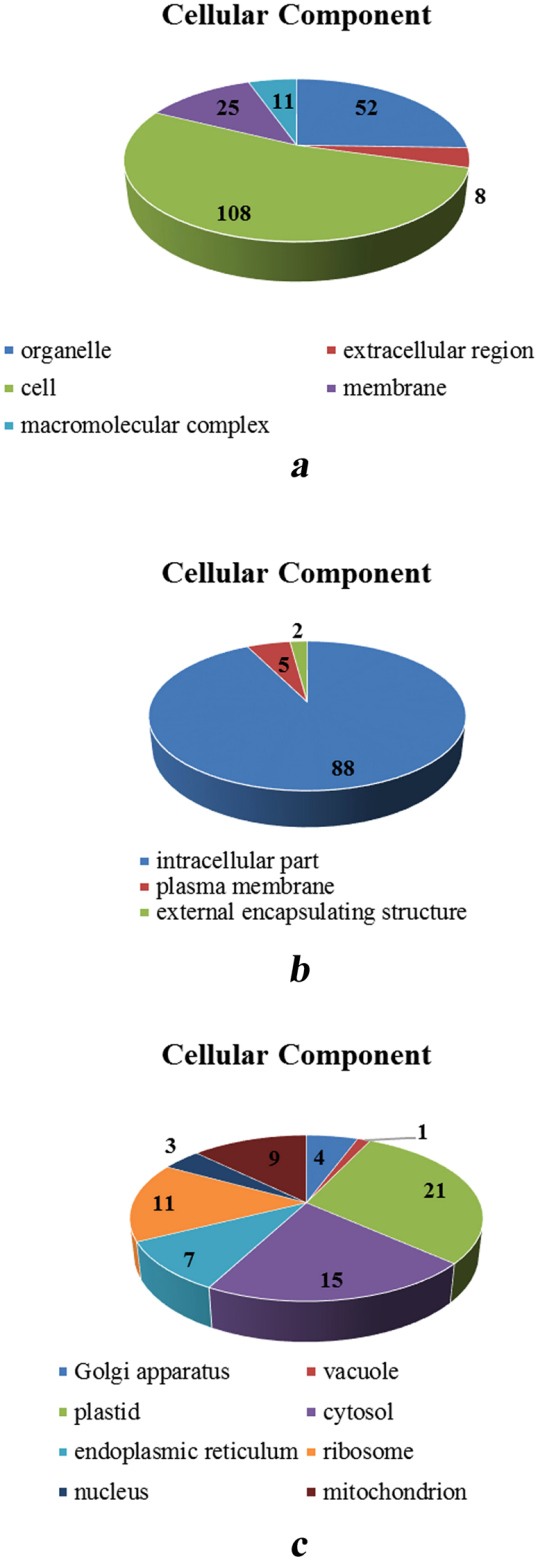
Cellular components are annotated in different GO level. (**a**) GO level 2, (**b**) GO level 5, (**c**) GO level 8. The number represents the number of differentially expressed proteins.

For wheat cultivar Jing411, differentially expressed proteins were mainly involved in protein metabolism (biological process), nucleotide binding (molecular function), and some intracellular substances (cellular component) at different levels of GO term. At GO level 2 in the cellular component, different numbers of proteins belong to different cellular components in different GO levels. For instance, at GO level 2, 102 differentially expressed annotated proteins belong to the cell, 8 belong to the extracellular region, 52 belong to the organelle, 25 belong to the membrane and 11 belong to the macromolecular complex (Fig. 4a). At GO level 5 or 8, differentially expressed proteins were annotated into more specific organism intracellular components (Fig. 4b,c). Therefore, through comparison, with a higher GO term level, the differential proteins were annotated with more specific components, and the number of proteins was lower. According to the different GO term levels, we can refine the specific proteins in cells, which belong to cellular components and are involved in biological processes and prossessed molecular functions. We can reveal the effect of heat stress on the grain filling process of wheat by analyzing the differentially expressed proteins that we are concerned with.

### KEGG pathway enrichment analysis for differentially expressed proteins

**Figure 5:**
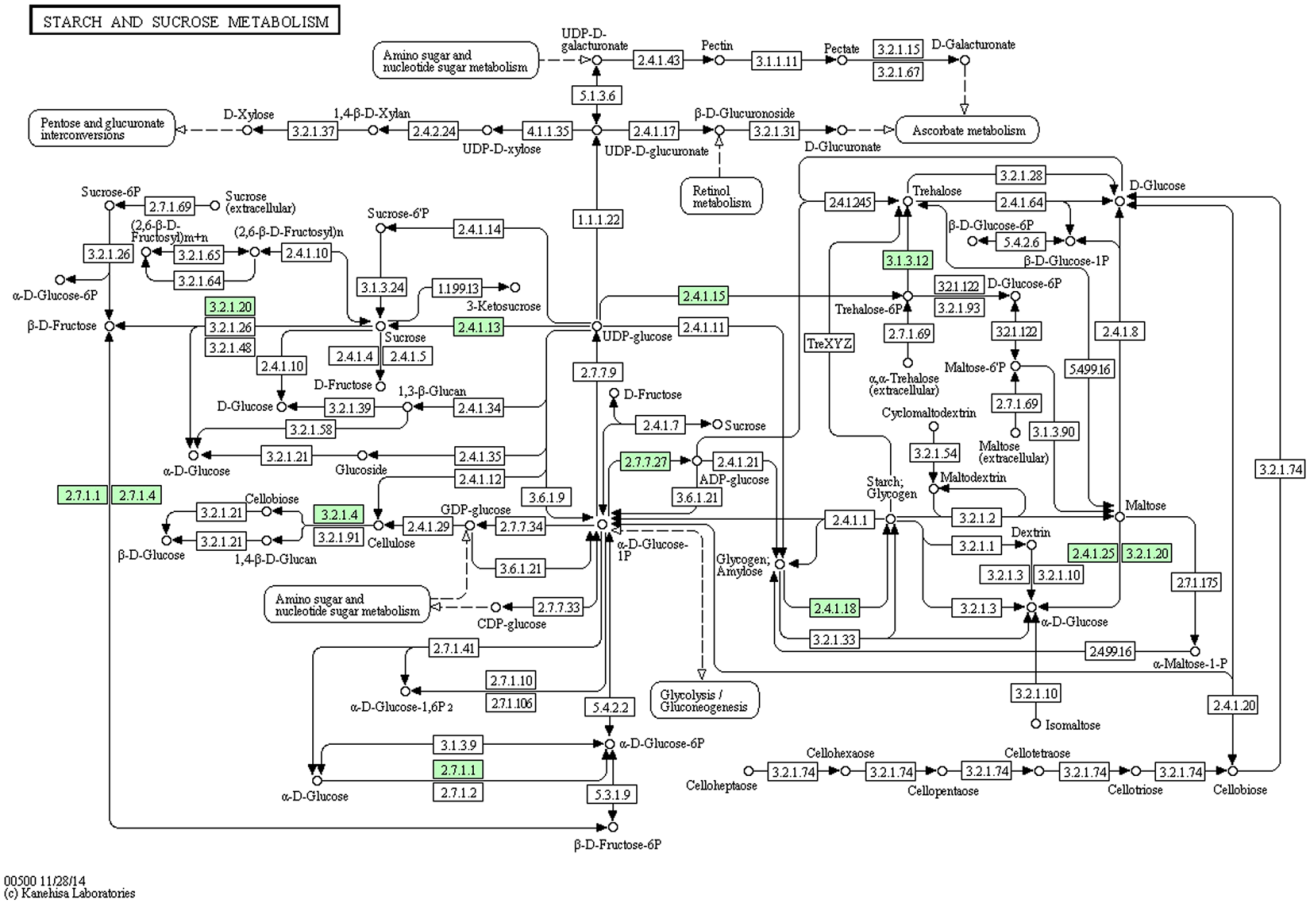
KEGG pathway annotation: starch and sucrose metabolism. The green number represents the differentially expressed proteins. These proteins are queried by http://www.kegg.jp/kegg-bin/show_pathway?ko00500+K00695.

**Figure 6:**
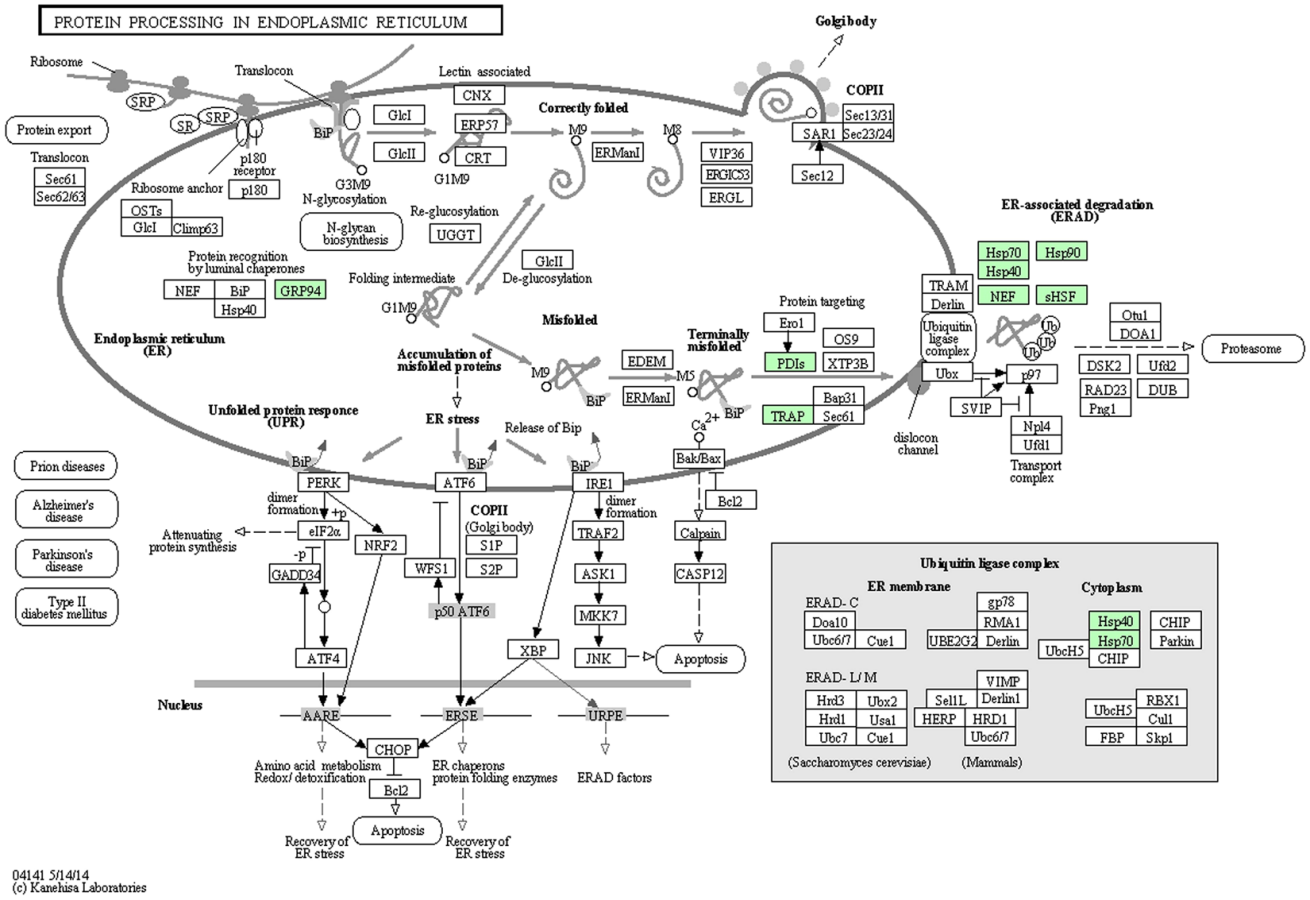
KEGG pathway annotation: protein processing in the endoplasmic reticulum. The green box represents the differentially expressed proteins. These proteins are queried by http://www.kegg.jp/kegg-bin/show_pathway?ko04141+K13993.

Many other metabolic pathways seem to be involved in the heat stress response; it should be noted that the heat stress effect observed here corresponds to the amount of protein, which does not necessarily mean a translational regulation. KEGG pathway enrichment analysis reveals the metabolic or signaling pathways in which differentially expressed proteins may be involved in the heat stress response. In this study, the set of differentially expressed proteins was primarily enriched in two pathways: protein processing in the endoplasmic reticulum (31 proteins) and starch and sucrose metabolism (13 proteins). These results suggest that heat stress may affect protein synthesis in the endoplasmic reticulum and the metabolism of starch and sucrose (Figs 5 and 6, Table S3, Enrichment). Further investigations of the molecular level of these pathways are required. It will be interesting to study the effect of a heat shock on the proteome of wheat grain, as this will contribute to understanding the molecular basis of the heat stress response.

### Analysis of protein-protein interaction networks

All proteins cannot perform their function alone, and they interact with other proteins to form a functional protein network structure; therefore, protein-protein interaction networks can reveal the roles and relative importance of proteins with respect to biological processes. In this study, proteins differentially expressed under heat stress were primarily involved in eight protein-protein interaction networks (Fig. 3). CBL3, ERG3, BRI1, and DPBF4 interacted with multiple proteins simultaneously; these proteins might participate in several biological processes during heat stress. Moreover, PDI, CLPB1, starch phosphorylase L-1, and PRXIIB interacted with a single protein.

Calcineurin B-like (CBL) genes are target genes of the calcium signaling pathway in plants and play an important role in stress signal transduction^31^. Tang *et al*.^32^ found that CBL2 and CBL3 serve as molecular links between calcium signaling and V-ATPase in *Arabidopsis*. Moreover, CBL family proteins are major regulators of intracellular ion balance^32^. *CBL1* serves as a positive regulator when plants are faced with salt or drought stress, while it acts as a negative regulator under chilling stress^33^. DRI1 is a receptor kinase that can transduce steroid signals through the plasma membrane^34^. CLPB1, known as *AtHSP101* in *Arabidopsis*, is the product of the *HOT1* and the CLP/HSP100 protein which has been studied most intensively in plants. CLPB1 is an HSP that accumulates rapidly under heat stress and has a vital regulatory effect on plant growth^35^. PrxIIB is usually found in the cytoplasm^35^ and has several functions in tobacco, including regulating ascorbate regeneration, maintaining the xanthophyll cycle, and influencing H_2_O_2_ production in peroxisomes^36^.

PDI catalyzes disulfide bond formation, reduction, and isomerization of newly synthesized proteins in the ER lumen. It also serves as a molecular chaperone that ensures that proteins in its subcellular compartment are folded properly^37^. In *Arabidopsis*, BRI1 is a transmembrane receptor kinase that responds to brassinolide. BRI1 protein abundance determines the degree of response to brassinolide and the number of brassinolide binding sites; BRI1 can mediate transmembrane signaling by steroids^34^. EHIR, a transcription inhibitor, participates in the ASF1-mediated silencing pathway^38^. Mazzoni *et al*.^39^ found that histone genes were down-regulated in *Saccharomyces cerevisiae* mutants overexpressing HIR1, suggesting a connection between HIR1 and apoptosis. EGR3 is an enzyme that binds to the endoplasmic reticulum membrane in eukaryotic organisms and catalyzes the formation of double bonds between C5 and C6 on the γ-7-sterol B ring, as well as the synthesis of oat sterol, ergosterol, and 7-lathosterol in yeast, plants, and vertebrates, respectively^40^. Moreover, eight differentially expressed proteins were involved in protein-protein interaction networks. Differential expression of the proteins identified in this study under heat stress may significantly influence the yield and quality of wheat grain.

## Materials and Methods

### Plant material and growth conditions

Winter wheat (*Triticum aestivum* L.) cultivar Jing411 was grown under natural conditions in 50 cm × 30 cm × 40 cm plastic boxes, and moved into an artificial climate chamber after flowering. The growing condition was at 25°C (12 h day)/18°C (12 h night) and air humidity was at 20–25%. Temperature of the treatment group was controlled at 40°C for two hours (12:00–14:00) from flowering to maturity, and the control group growing condition was at 25°C (12 h day)/18°C (12 h night) and air humidity of 20–25%.

### SDS-sedimentation analysis

Wheat flour samples (2 g) were suspended in 16.7 mL H_2_O (containing 100 ppm bromophenol blue) and incubated at room temperature (≈25°C) for 5 min; 16.7 mL of SDS solution (2% SDS, 0.012 mol L^−1^ lactic acid) was then added to each sample, and each was again incubated at room temperature for an additional 5 min. Sedimentation volumes were recorded every 5 min for a total of 20 min.

### RNA Extraction and qRT-PCR

The wheat grain tissues from both the normal growth and heat-treatment were collected at 5, 10, 15, and 20 DPA. Wheat growth conditions followed plant material and growth conditions. Total RNA was extracted using an RNA pure Plant Kit (TIANGEN). cDNA was synthesized from total RNA using a PrimeScriptTM RT reagent Kit and oligo (dT) primers (Takara). The gene-specific primers of the target genes and Actin gene were designed using DNAMAN software; the primer sequences are listed in Table S4. qRT-PCR was conducted with an Applied Biosystems 7500 Real-Time PCR System (ABI, USA). The reaction conditions are as follows PrimeScriptTM RT reagent Kit. The expression levels of target genes were calculated using the 2^−ΔΔCt^ method, all reactions were performed in triplicate, and each experiment included three independent biological repetitions.

### Protein extraction

Grain samples were ground into fine powder in liquid nitrogen using a mortar and pestle, and 2 g samples were extracted for 2 h with 3 ml extraction buffer (50 mM Tris-HCl, pH 8.0, 0.1 M KCl, 5 mM EDTA, 30% sucrose) containing 1 mM PMSF. After centrifuging for 15 min at 13,000 rpm, supernatants were transferred to new tubes. 40 mL cold TCA/acetone solution was added to the supernatants and stored at −20°C overnight, followed by centrifuging at 7,830 rpm for 30 min, and the supernatant was subsequently removed. 40 mL acetone was added for pre-cooling and centrifuged at 7,830 rpm, 4°C for 30 min, and this procedure was repeated until the acetone was completely colorless. The precipitate was dried at room temperature. Protein samples were incorporated into 500 μL STD buffer (4% SDS, 1 mM DTT, 150 mM Tris-HCl, pH 8.0), incubated at room temperature, shocked for 1 h, and then centrifuged at 13,400 rpm for 30 min. Protein concentrations were determined by the bicinchoninic acid (BCA) method.

### Protein Digestion and iTRAQ Labeling

Protein digestion was performed according to the FASP procedure described by Wisniewski *et al*.^41^, and the resulting peptide mixture was labeled using the 4-plex/8-plex iTRAQ reagent according to the manufacturer’s instructions (Applied Biosystems). Briefly, 200 μg of proteins for each sample was incorporated into 30 μL STD buffer (4% SDS, 100 mM DTT, 150 mM Tris-HCl, pH 8.0). The detergent, DTT, and other low-molecular-weight components were removed using UA buffer (8 M Urea, 150 mM Tris-HCl, pH 8.0) by repeated ultrafiltration (Microcon units, 30 kD). Then, 100 μL 0.05 M iodoacetamide in UA buffer was added to block reduced cysteine residues and the samples were incubated for 20 min in darkness. The filters were washed with 100 μL UA buffer three times and then with 100 μL DS buffer (50 mM triethylammonium bicarbonate at pH 8.5) twice. Finally, the protein suspensions were digested with 2 μg trypsin (Promega) in 40 μL DS buffer overnight at 37°C, and the resulting peptides were collected as a filtrate. The peptide content was estimated by UV light spectral density at 280 nm using an extinctions coefficient of 1.1 of 0.1% (g/L) solution which was calculated on the basis of the frequency of tryptophan and tyrosine in vertebrate proteins.

For labeling, each iTRAQ reagent was dissolved in 70 μL of ethanol and added to the respective peptide mixture. The samples were labeled (Sample1)-114, (Sample2)-115, (Sample3)-116, and (Sample4)-117 and were multiplexed and vacuum dried.

### Peptide Fractionation with Strong Cation Exchange (SCX) Chromatography

iTRAQ-labeled peptides were fractionated by SCX chromatography using the AKTA Purifier system (GE Healthcare). The dried peptide mixture was reconstituted and acidified with 2 mL buffer A (10 mM KH_2_PO_4_ in 25% of ACN, pH 2.7) and loaded onto a polysulfoethyl 4.6 × 100 mm column (5 µm, 200 Å, PolyLC Inc, Maryland, U.S.A.). The peptides were eluted at a flow rate of 1 mL/min with a gradient of 0–10% buffer B (500 mM KCl, 10 mM KH_2_PO_4_ in 25% of ACN, pH 2.7) for 2 min, 10–20% buffer B for 25 min, 20–45% buffer B for 5 min, and 50–100% buffer B for 5 min. The elution was monitored by absorbance at 214 nm, and fractions were collected every 1 min. The collected fractions (approximately 30 fractions) were finally combined into 10 pools and desalted on C18 Cartridges (Empore™ SPE Cartridges C18 (standard density), bed I.D. 7 mm, volume 3 mL, Sigma). Each fraction was concentrated by vacuum centrifugation and reconstituted in 40 µL of 0.1% (v/v) trifluoroacetic acid. All samples were stored at −80 °C until LC-MS/MS analysis.

### Liquid Chromatography (LC) - Electrospray Ionization (ESI) Tandem MS (MS/MS) Analysis by Q Exactive

Experiments were performed on a Q Exactive mass spectrometer that was coupled to Easy nLC (Proxeon Biosystems, now Thermo Fisher Scientific). 10 μL of each fraction was injected for nanoLC-MS/MS analysis. The peptide mixture (5 μg) was loaded onto the C18-reversed phase column (Thermo Scientific Easy Column, 10 cm long, 75 μm inner diameter, 3μm resin) in buffer A (0.1% Formic acid) and separated with a linear gradient of buffer B (80% acetonitrile and 0.1% Formic acid) at a flow rate of 250 nL/min controlled by IntelliFlow technology for 140 min. MS data were acquired using a data-dependent top10 method dynamically selecting the most abundant precursor ions from the survey scan (300–1800 m/z) for HCD fragmentation. Determination of the target value is based on predictive Automatic Gain Control (pAGC). Dynamic exclusion duration was 60 s. Survey scans were acquired at a resolution of 70,000 at m/z 200 and resolution for HCD spectra was set to 17,500 at m/z 200. Normalized collision energy was 30 eV and the underfill ratio, which specifies the minimum percentage of the target value likely to be reached at maximum fill time, was defined as 0.1%. The instrument was run with peptide recognition mode enabled.

### Sequence Database Searching and Data Analysis

MS/MS spectra were searched using MASCOT engine (Matrix Science, London, UK; version 2.2) embedded into Proteome Discoverer 1.3 (Thermo Electron, San Jose, CA.) against uniprot_Triticum_aestivnm_106567_20141024.fasta (106567 sequences, downloaded on October 24th, 2014) and the decoy database. For protein identification, the following options were used. Peptide mass tolerance = 20 ppm, MS/MS tolerance = 0.1 Da, Enzyme = Trypsin, Missed cleavage = 2, Fixed modification: Carbamidomethyl (C), iTRAQ4/8 plex(K), iTRAQ4/8plex(N-term), Variable modification: Oxidation (M), FDR ≤ 0.01.

### The analysis method for gathering biological information about the differentially expressed proteins selected

The sequence data of the selected differentially expressed proteins were retrieved in batches from UniProtKB database (Release 2014_12) in FASTA format. The retrieved sequences were locally searched against SwissProt database (mouse) using the NCBI BLAST+ client software (ncbi-blast-2.2.28+ -win32. exe) to find homologue sequences from which the functional annotation can be transferred to the studied sequences. In the present work, the top 10 blast hits with an E-value less than 1e^−3^ for each query sequence were retrieved and loaded into Blast2GO^42^ (Version 2.8.0) for GO^43^ mapping and annotation. The sequences without BLAST hits and un-annotated sequences were then selected to undergo an InterProScan^44^ against EBI databases to retrieve functional annotations of protein motifs and merge the InterProScan GO terms to the annotation set. Following annotation and annotation augmentation steps, the studied proteins were blasted against KEGG GENES (sp.) to retrieve their KOs and were subsequently mapped to pathways in KEGG^45^. The protein interaction data of the studied proteins were retrieved from IntAct molecular interaction database^46^ by their gene symbols. The results were downloaded in the XGMML format and imported into Cytoscape^47^ for further analysis.

## Electronic supplementary material

Supplementary Info

Supplementary 2

Supplementary data 3 

Supplementary data 4 
