# Supplementary material for: Differential effects of a post-anthesis heat stress on wheat (Triticum aestivum L.) grain proteome determined by iTRAQ
Source: Sci Rep. 2017 Jun 14;7:3468. doi: 10.1038/s41598-017-03860-0 (PMC5471245; doi:10.1038/s41598-017-03860-0)
Supplement: Supplementary file 1 — Supplementary Info [file 41598_2017_3860_MOESM1_ESM.doc]

**Differential effects of a post-anthesis heat stress on wheat (*Triticum aestivum* L.) grain proteome determined by iTRAQ**

Yufeng Zhang^1,2^, Jiajia Pan^1^, Xiuwen Huang^1^, Dandan Guo^1^, Hongyao Lou^1^, Zhenghong Hou^1^, Meng Su^1^, Rongqi Liang^1^, Chaojie Xie^1^, Mingshan You^1^ and Baoyun Li^*1^

**Supplementary Data**

**Supplemental Table S1 Protein significant analysis table.**

Sheet 1 shows the protein expression profile as indicated by iTRAQ in Jing411 under heat stress.

Sheet 2 shows the protein expression profile change of Jing411 under heat stress.

**Table S2 GO annotation and enrichment analysis for differentially expressed proteins.**

Table S2 contains TopBlastHits, protein2GO, BP, MF, CC, Level2_BP, Level2_MF and Level2_CC. BP: biological Process; MF: molecular Function; CC: Cellular Component.

**Table S3 KEGG pathway annotation and KEGG enrichment analysis for differentially expressed proteins.**

Table S3 contains query2map, map2query, TopMapStat and enrichment.

**Table S4 Primer pairs used for qRT-PCR in this study.**

| Primer Names | sequence |
| --- | --- |
| *HSP* 90-F | CATCAACACCTTCTACTCCA |
| *HSP* 90-R | GCTTGCTCTTGTCTGTCA |
| *LMW-GS*-F | TATCCTGCCAACGATGTG |
| *LMW-GS*-R | CTTTATTTGTCACCGCTGC |
| *SBEIIb*-F | GACATTGATGAACACGAAGG |
| *SBEIIb*-R | AGCCCATTCTCGGTAAGTG |
| *ADPG-PPase*-F | TCACATTCACCGCACCTA |
| *ADPG-PPase*-R | TCCTCAAGCACCCAGATA |

**Fig. S1 (a) Grain shapes of wheat cultivar Jing411 under heat stress (T) and normal growth conditions (CK). (b) SDS-sedimentation of wheat cultivars under heat stress and normal conditions**. **(c) Differences in the grain traits of wheat cultivar Jing411 under heat stress (T) and normal growth conditions (CK).** (1000-grain weight: determined by three biological replicates, each of which included three measurements of 1,000 randomly selected grains; grain width and length: determined by three biological replicates, each of which included three measurements of 100 randomly selected grains. Note: * denotes a significant difference (P < 0.05) and ** denotes an extremely significant difference (P < 0.01). Normal growth conditions (CK) and heat stress (T), respectively.

**Fig. S2 Correlational analysis of protein expression levels in the high temperature treatment group (a) and normal treatment group (b) in cultivar Jing 411. (a)** A114, B114, and C114 represent three independent biological repetitions of proteins under normal conditions. (b) A115, B115, and C115 represent three independent biological repetitions of proteins under high temperature conditions.

**Fig. S3 Sequence alignment similarity distribution.** The identified proteins were blasted against the NCBI nr database using NCBI BLAST+ software (ncbi-blast-2.2.28+-win32.ext).

**Fig. S4 GO-level distribution.** P: Biological Process, F: Molecular Function, C: Cellular Components

**Fig. S5 Variation in the transcript levels of** **differentially expressed proteins corresponding to genes in normal growth conditions (CK) and heat stress (T) in wheat grain.** (a) Adenosine diphosphoglucose pyrophosphorylase (*ADPG-PPase*), (b) Heat shock protein 90 (*HSP90*), (c) Starch branching enzyme IIb (*SBEIIb*), (d) Low-molecular-weight glutenin subunits (*LMW-GS*).


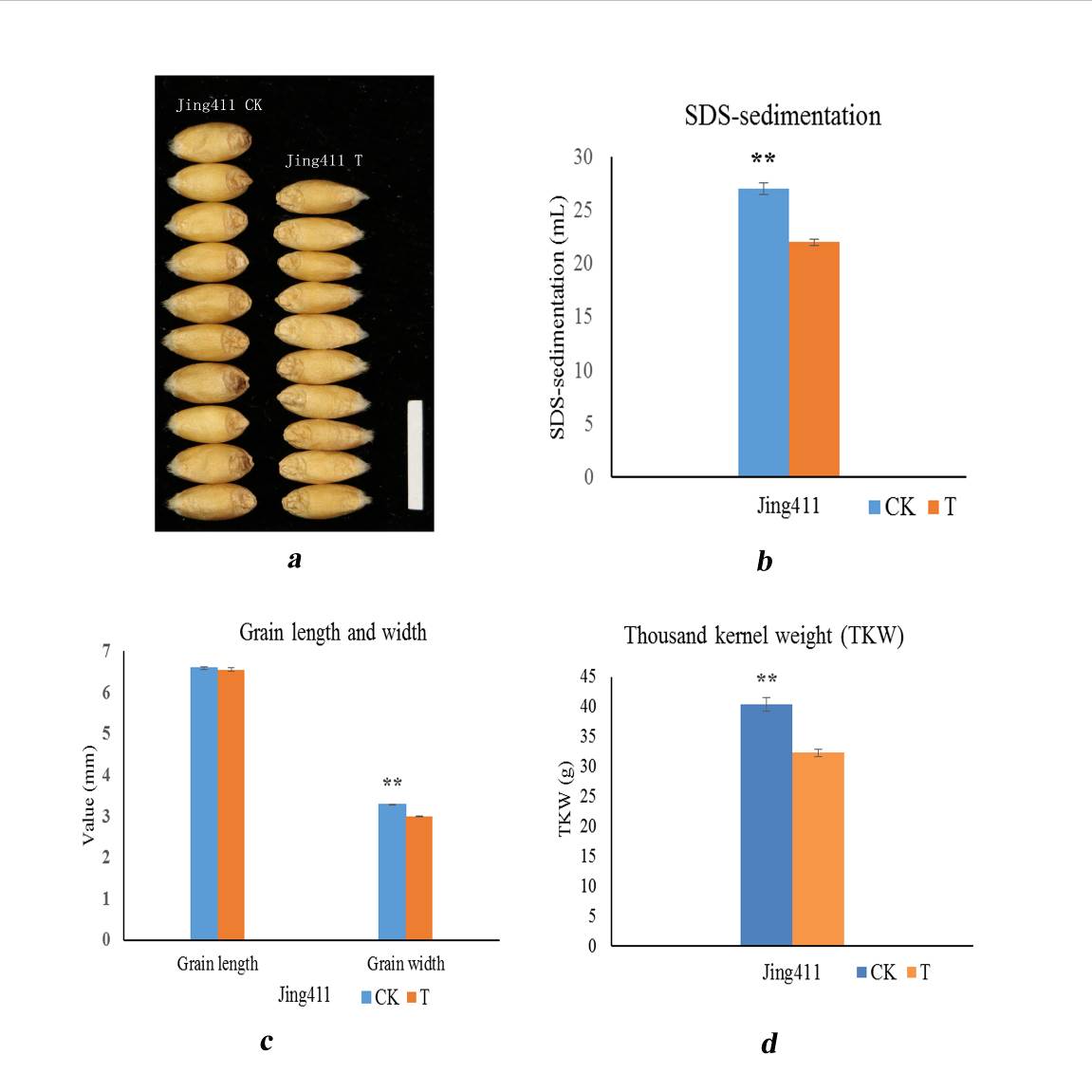


**Fig. S1**


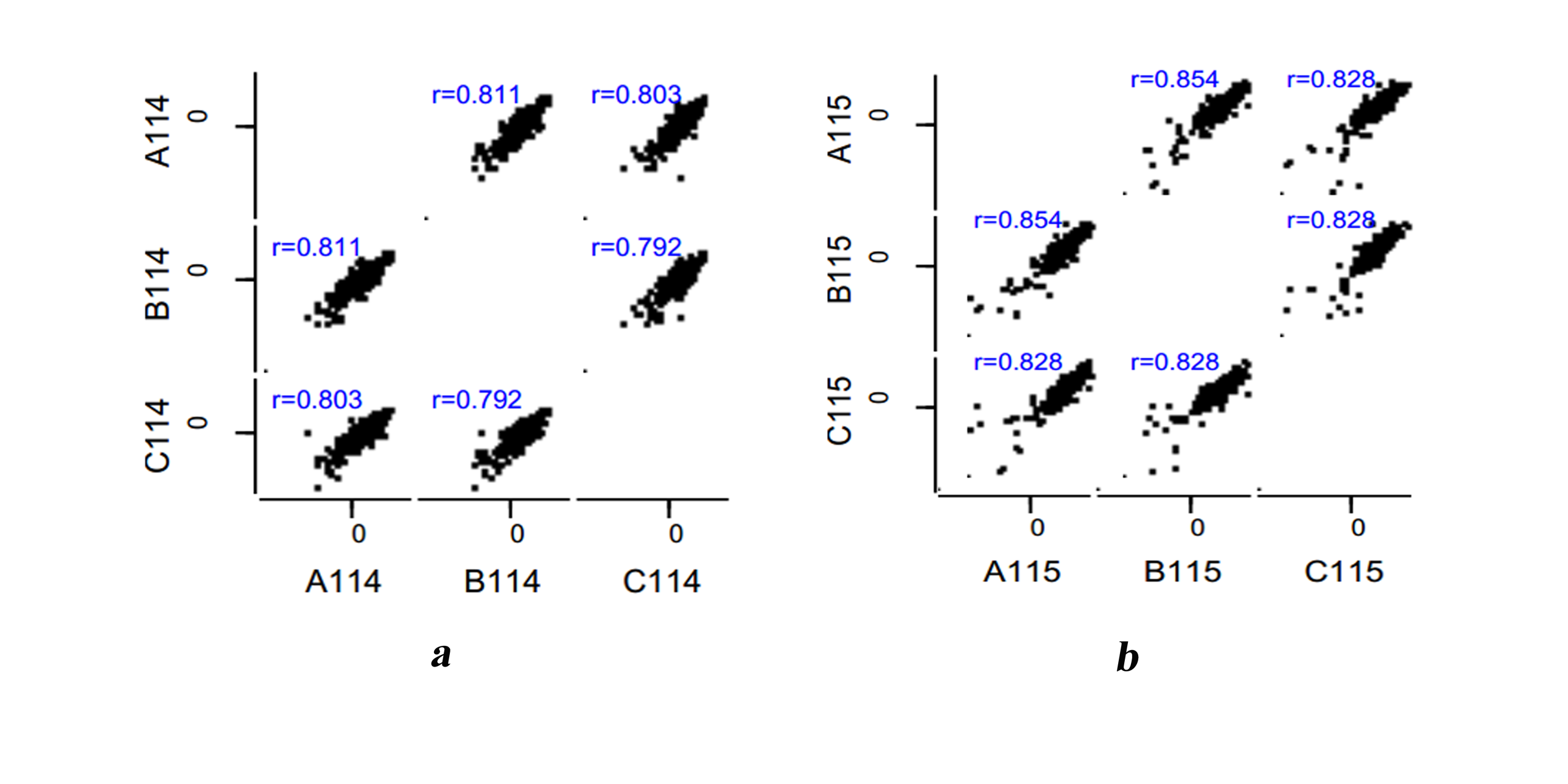


**Fig. S2**

**
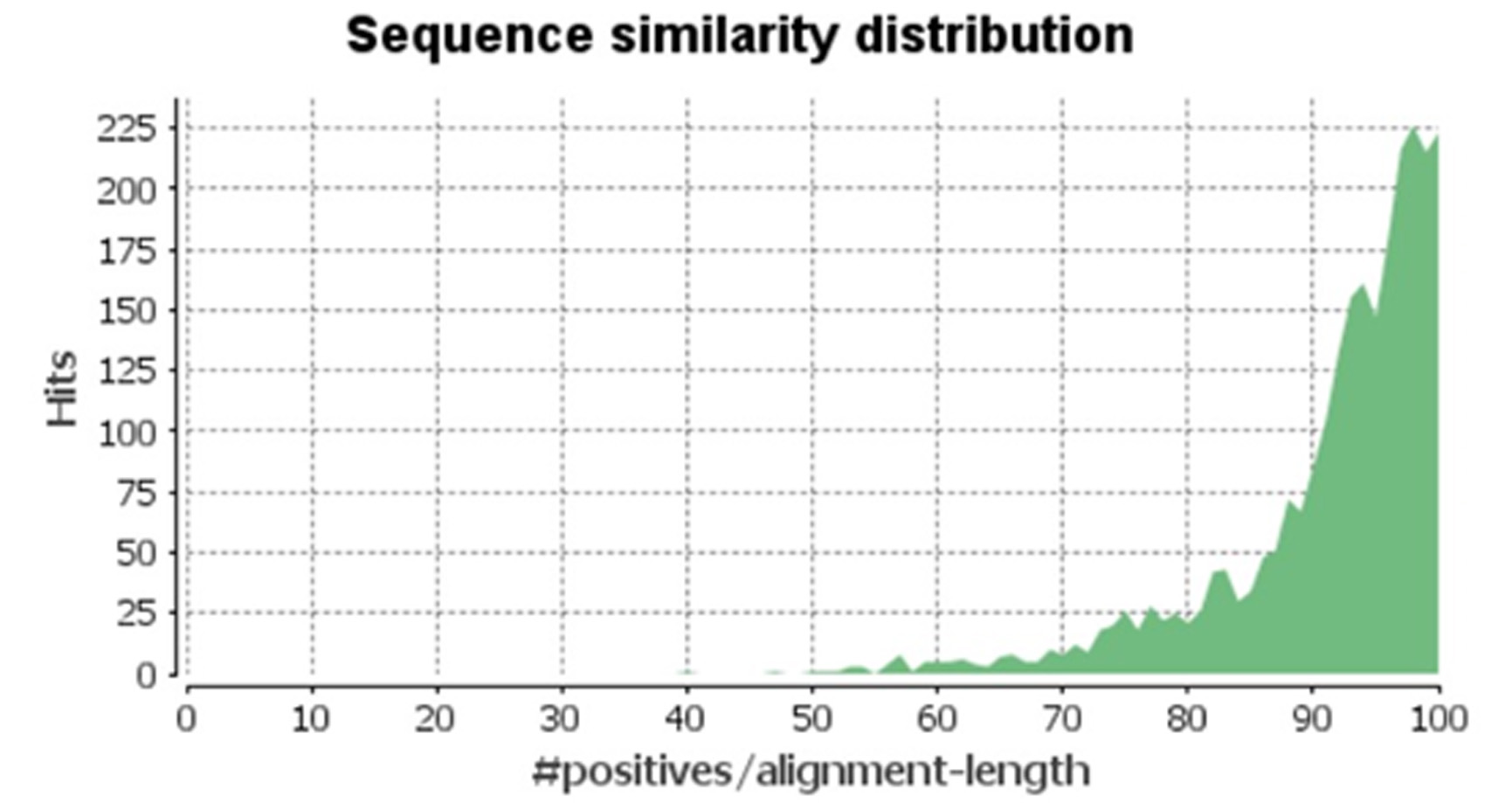
**

**Fig. S3**

**
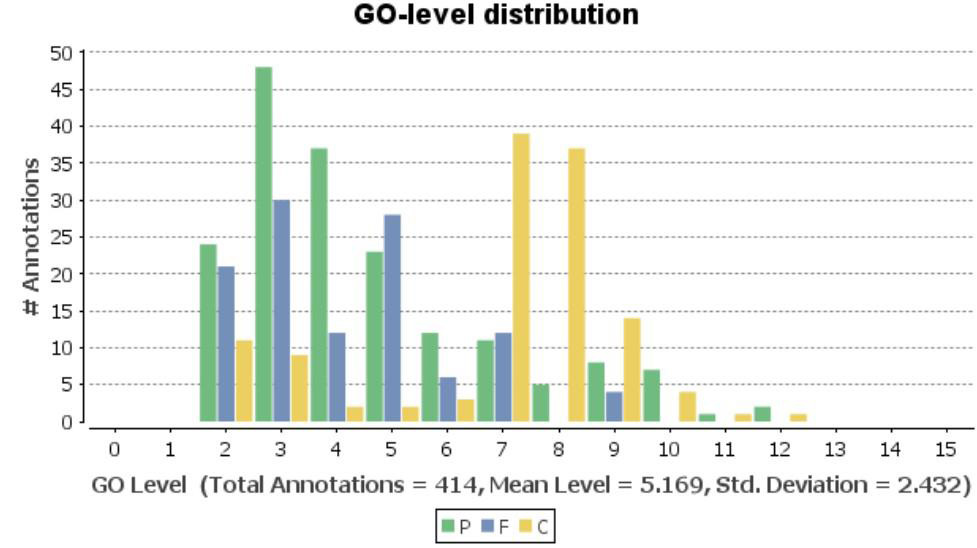
**

**Fig. S4**

**
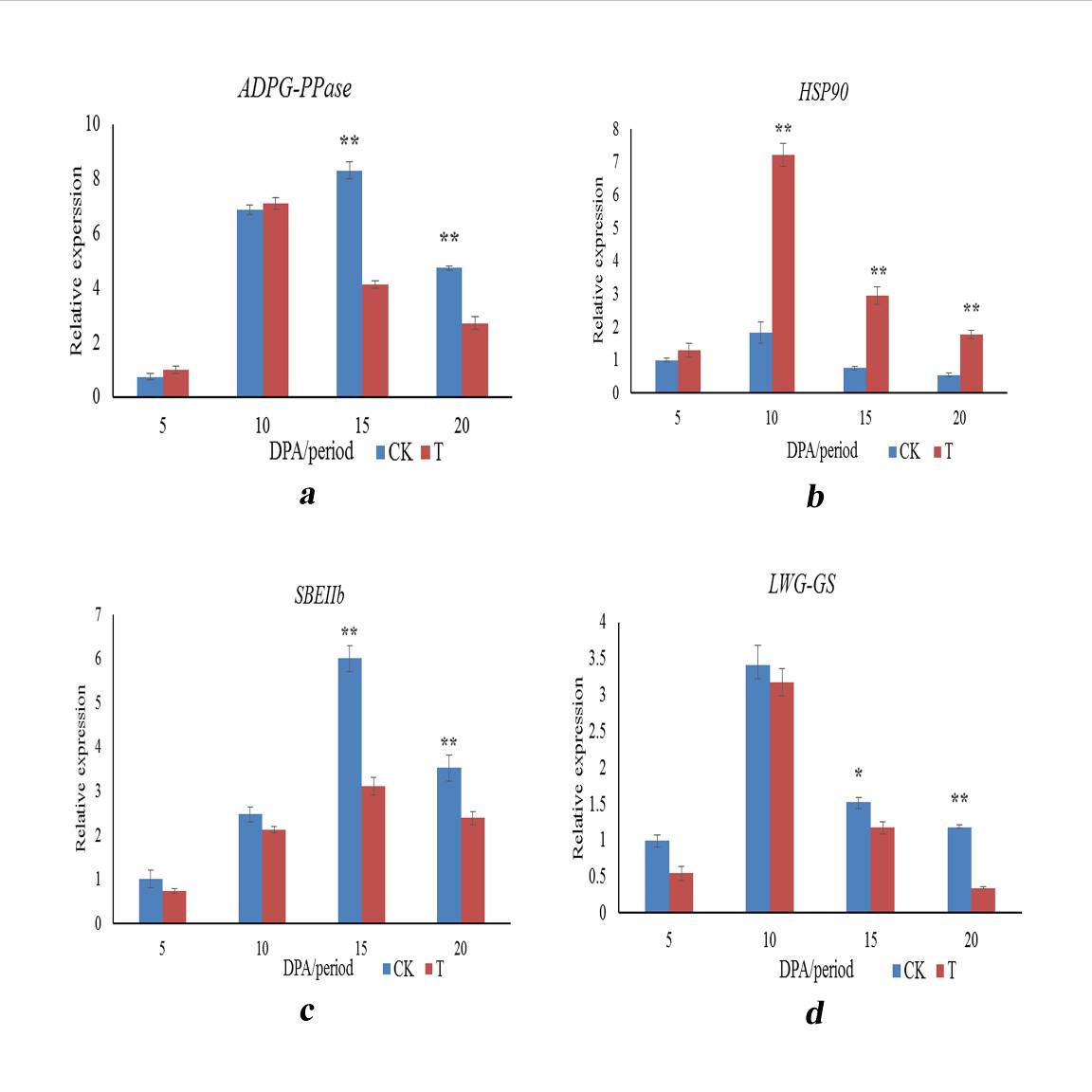
**

**Fig. S5**
